# P2Y_6_ Receptor Activation Promotes Inflammation and Tissue Remodeling in Pulmonary Fibrosis

**DOI:** 10.3389/fimmu.2017.01028

**Published:** 2017-08-22

**Authors:** Tobias Müller, Susanne Fay, Rodolfo Paula Vieira, Harry Karmouty-Quintana, Sanja Cicko, Cemil Korcan Ayata, Gernot Zissel, Torsten Goldmann, Giuseppe Lungarella, Davide Ferrari, Francesco Di Virgilio, Bernard Robaye, Jean-Marie Boeynaems, Eduardo R. Lazarowski, Michael R. Blackburn, Marco Idzko

**Affiliations:** ^1^Department of Pneumology, University Medical Center Freiburg, Freiburg, Germany; ^2^Division of Pneumology, University Hospital RWTH Aachen, Aachen, Germany; ^3^Department of Biochemistry and Molecular Biology, University of Texas, Houston, TX, United States; ^4^Clinical and Experimental Pathology, Research Center Borstel, Borstel, Germany; ^5^Department of Physiopathology and Experimental Medicine, University of Siena, Siena, Italy; ^6^Department of Morphology, Surgery and Experimental Medicine, Section of Pathology, Oncology and Experimental Biology, University of Ferrara, Ferrara, Italy; ^7^IRIBHM and Erasme Hospital, Université Libre de Bruxelles, Brussels, Belgium; ^8^Cystic Fibrosis Research Center, Marsico Lung Institute, University of North Carolina, Chapel Hill, NC, United States

**Keywords:** pulmonary fibrosis, inflammation, purinergic receptors, uridine-5′-diphosphate, animal model, bleomycin

## Abstract

Idiopathic pulmonary fibrosis (IPF) is a disease with a poor prognosis and very few available treatment options. The involvement of the purinergic receptor subtypes P2Y_2_ and P2X_7_ in fibrotic lung disease has been demonstrated recently. In this study, we investigated the role of P2Y_6_ receptors in the pathogenesis of IPF in humans and in the animal model of bleomycin-induced lung injury. P2Y_6_R expression was upregulated in lung structural cells but not in bronchoalveolar lavage (BAL) cells derived from IPF patients as well as in animals following bleomycin administration. Furthermore, BAL fluid levels of the P2Y_6_R agonist uridine-5′-diphosphate were elevated in animals with bleomycin-induced pulmonary fibrosis. Inflammation and fibrosis following bleomycin administration were reduced in P2Y_6_R-deficient compared to wild-type animals confirming the pathophysiological relevance of P2Y_6_R subtypes for fibrotic lung diseases. Experiments with bone marrow chimeras revealed the importance of P2Y_6_R expression on lung structural cells for pulmonary inflammation and fibrosis. Similar effects were obtained when animals were treated with the P2Y_6_R antagonist MRS2578. *In vitro* studies demonstrated that proliferation and secretion of the pro-inflammatory/pro-fibrotic cytokine IL-6 by lung fibroblasts are P2Y_6_R-mediated processes. In summary, our results clearly demonstrate the involvement of P2Y_6_R subtypes in the pathogenesis of pulmonary fibrosis. Thus, blocking pulmonary P2Y_6_ receptors might be a new target for the treatment of IPF.

## Introduction

Idiopathic pulmonary fibrosis (IPF) is deadly lung disease, which is believed to arise from aberrant proliferation of fibrous tissue following tissue injury. Different cell types including inflammatory cells, lung fibroblasts, and abnormally activated alveolar epithelial cells have been shown to play a prominent role in IPF pathophysiology. Histologically characteristic changes such as myofibroblast accumulation in the alveolar airspaces and excessive deposition of extracellular matrix components can be observed. Though some progress has been made, very few treatment options are available for IPF, at least partly due to the lack of knowledge about the processes regulating the progression of tissue injury to tissue fibrosis (1–3).

Nucleotides such as adenosine-5′-triphosphate (ATP), adenosine-5′-diphosphate, uridine-5′-triphosphate, or uridine-5′-diphosphate (UDP) are released into the extracellular space under various conditions, including inflammation or hypoxia (4–7). The cellular effects of extracellular nucleotides are mediated *via* P2 purinergic receptors (P2Rs) which can be subdivided into G protein-coupled P2Y (P2Y_1_, P2Y_2_, P2Y_4_, P2Y_6_, P2Y_11_–P2Y_14_) and P2X receptors (P2X_1_–P2X_7_) which are ligand-gated ion channels (8). Functional P2R are expressed on both inflammatory and lung structural cells and P2R activation is associated with a broad range of cellular responses, including migration, cytokine secretion, release of reactive oxygen species, or apoptosis (5, 8, 9). The involvement of specific P2R subtypes in the pathophysiology of lung diseases, e.g., bronchial asthma or chronic obstructive pulmonary disease is well established (4, 6, 9–11). Increased extracellular ATP levels have been measured in the bronchoalveolar lavage (BAL) fluid derived from IPF patients or animals with bleomycin-induced pulmonary fibrosis, whereas deficiency in distinct P2R subtypes such as P2X_7_R or P2Y_2_R was associated with reduced inflammation and fibrosis following bleomycin administration (12–14). Nevertheless, as the expression of purinergic receptors is widespread, the involvement of more than one P2R subtype is likely.

In contrast to other P2R subtypes, the P2Y_6_ receptor is preferentially activated by UDP and not by ATP (9, 15, 16). P2Y_6_ receptors have been linked with the pathophysiology of inflammatory bowel disease, vascular inflammation, and cardiac fibrosis (17–19). Previously, we were able to demonstrate that P2Y_6_ receptors contribute to acute and chronic allergic airway inflammation (9). However, the role of this receptor subtype in the context of fibrotic lung disease has not been investigated in detail yet.

## Materials and Methods

### Patient Materials

Bronchoalveolar lavage fluids were collected from patients undergoing bronchoscopy during the diagnostic workup of IPF or from healthy volunteers. In addition, surgical lung biopsies derived from IPF patients or tumor free margins of lung cancer resections as control were used. IPF was diagnosed according to published criteria (20). The study was approved by the local ethics committee (ethics committee at the University Medical Center Freiburg).

### Animals

P2Y_6_R-deficient and wild-type (WT) animals (both on C57BL/6 background) were bred at the University Freiburg. All experiments were approved by the local animal ethics committee (Regierungspräsidium Freiburg).

### Generation of Chimeric Animals with P2Y_6_R Deficiency on Hematopoietic or Structural Cells

Wild-type or P2Y_6_R-deficient mice were given 5 × 10^6^ bone marrow cells derived from WT or P2Y_6_R-deficient animals intravenously after irradiation with 900 cGy (2× 450 cGy). The following donor/recipient pairs were combined: WT BM → WT, P2Y_6_R^−/−^ BM → WT, WT BM → P2Y_6_R^−/−^ and P2Y_6_R^−/−^ BM → P2Y_6_R^−/−^.

### Bleomycin Model of Pulmonary Fibrosis

Male C57BL/6 or P2Y_6_R-deficient animals (6–8 weeks old) were anesthetized *via* intraperitoneal injection with ketamine/xylazine (2/0.1 mg) and received an intratracheal (i.t.) injection of bleomycin (80 µl; 0.5 mg/ml). In some experiments, animals were treated intratracheally with the P2Y_6_R antagonist MRS2578 or vehicle in either a prophylactic (d0, d5, d10 after bleomycin application) or therapeutic protocol (from day 14 after bleomycin application, for three times a week). Animals were killed at different time points *via* intraperitoneal (i.p.) injection of pentobarbital as indicated. BAL was performed with 3× 1 ml of Ca^2+^- and Mg^2+^-free PBS supplemented with 0.1 mM sodium EDTA, followed by lung resection and storage in OCT freezing medium. BAL cells were counted using a hemocytometer, and differential cell counts were done by fluorescence-activated cell sorter (FACS) analysis, as described previously (21). Briefly BAL cells were stained for 30 min with anti-Ly6 B FITC (Serotec, Düsseldorf, Germany), anti-F4/80 Pe (eBioscience, San Diego, CA, USA), anti-CD3 and anti-CD19 cy-chrome (eBioscience, San Diego, CA, USA), and anti-CD45 APC (ImmunoTools, Friesoythe, Germany) in PBS containing 0.5% BSA followed by FACS analysis. Frozen lung sections were stained with hematoxylin/eosin for histological analysis. Lung slides were also stained with picrosirius red for collagen quantification. Therefore, frozen lung sections were incubated in picrosirius red solution for 1 h. After washing with water, tissue sections were stained with hematoxylin for 5–10 s. Slides were washed with running tap water and dehydrated in 70%, 90%, and absolute ethanol, followed by xylene. Entellan (Merck) was used to mount the coverslip. Images were obtained using Axio Lab.A1 microscope (Zeiss) with 200× magnification and AxioCam ICc1 (Zeiss). Collagen quantification was made with ImageJ software (21).

### Mediator Measurements in BALF

Uridine-5′-diphosphate concentrations in BALF were measured by HPLC as previously described (22). BALF collagen content was measured by Sircol assay (Biocolor, Carrickfergus, UK). The concentration of cytokines in the BAL fluid was determined by ELISA (R&D Systems, Minneapolis, MN, USA).

### PCR

Total RNA from human or murine lung tissue was isolated using RNeasy mini-kits (Qiagen, Hilden, Germany) according to the manufacturer’s recommendations followed by reverse transcription using Stratascript reverse transcriptase (Stratagene, La Jolla, CA, USA) and random primers (Invitrogen, Karlsruhe, Germany). Quantitative PCR was performed with Taqman Universal PCR Mastermix (Applied Biosystems, Foster City, CA, USA) and pre-formulated primers and probe mixes (Assay on Demand; Applied Biosystems). PCR was performed on a thermal cycler (iCycler; Bio-Rad, Hercules, CA, USA). PCR amplification of the housekeeping gene encoding glyceraldehyde 3-phosphate dehydrogenase was performed during each run for each sample to allow normalization between samples.

### Isolation of Primary Murine Lung Fibroblasts

Lungs of P2Y_6_R-deficient or WT animals were excised and cut in small pieces, followed by digestion with collagenase. Single cell suspensions were obtained by passing through a cell strainer. Cells were plated on six-well plates in cell culture medium at 37°C, 5% CO_2_ in a humidified atmosphere. Medium was changed every 2 or 3 days. Cells were trypsinized after 14 days and used for experiments. Purity of isolated cells was assessed by microscopy (typical morphology of fibroblasts) (23).

### Isolation of Primary Human Lung Fibroblasts

Primary lung Fibroblasts were isolated as described previously (23). Briefly, histologically normal lung tissue was obtained from resected lung specimens from subjects with central lung cancers or from patients with benign tumors. Lung tissue was sliced and washed. The washed slices were placed into six-well plates containing Quantum (PAA, Pasching, Austria) and 1% penicillin/streptomycin solution. The slices were maintained in the medium at 37°C in a 5% CO_2_ incubator for 2–3 weeks. The cells that grew from these tissue slices were serially passaged several times to yield pure populations of lung FBRs. The fibroblast cell lines were assessed by cell morphology, immunostaining for vimentin, and FACS analysis.

### Proliferation of Lung Fibroblasts

Fibroblasts (2 × 10^5^ cells/well) were seeded into cell culture plates and stimulated with UDP or vehicle (sterile phosphate-buffered saline). Higher concentrations of FCS were used as a positive control. After 48 h, cells were trypsinized and counted. Results are shown as proliferation index, calculated as the number of cells stimulated with UDP divided by the number of vehicle-treated cells.

### Measurement of S6 Phosphorylation

Fibroblasts were stimulated with the indicated nucleotides or vehicle. Cells were lysed with RIPA buffer at different time points. Cell lysates were analyzed by immunoblot using antibodies for phospho-S6 (Cell Signaling Technology, USA) and Actin (Sigma, Steinheim, Germany).

### Statistical Analysis

If not stated otherwise, groups were compared using one-way ANOVA, followed by Bonferroni comparison test (GraphPad Prism Software, San Diego, CA, USA). A *p*-value <0.05 was considered as significant.

## Results

**Figure 1:**
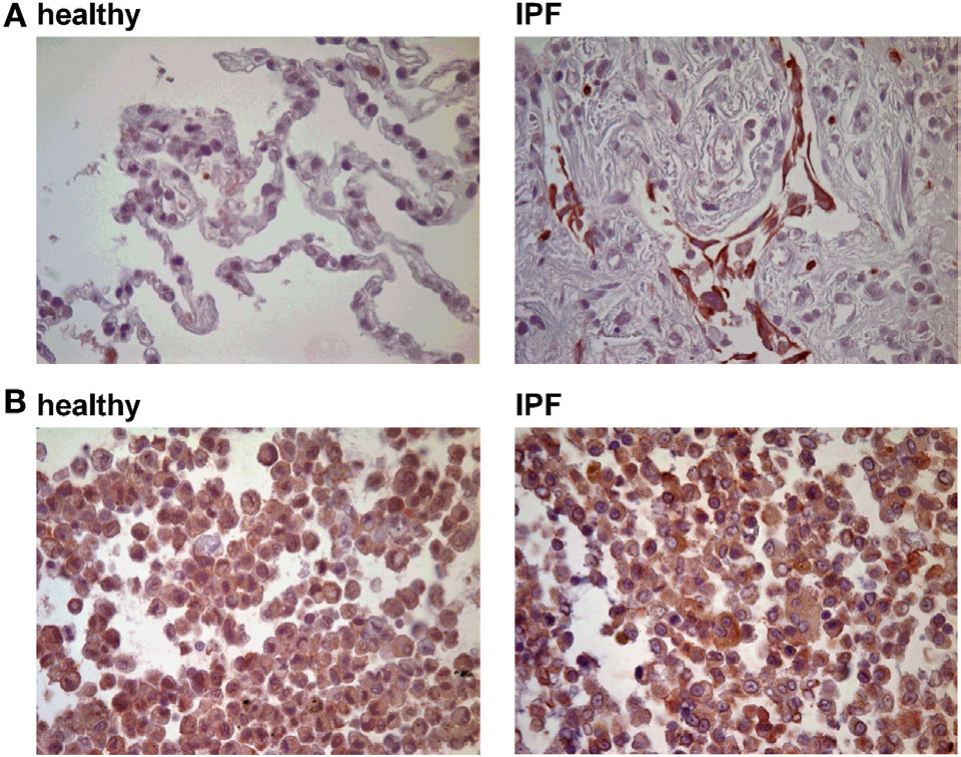
P2Y_6_R expression in idiopathic pulmonary fibrosis (IPF). **(A)** Immunohistochemical stainings for P2Y_6_R expression were performed with lung tissue derived from IPF patients (*n* = 5) or controls (tumor-free margins from lung cancer resections; *n* = 5). **(B)** Bronchoalveolar lavage cells derived from IPF patients (*n* = 5) and healthy volunteers (*n* = 5) were stained for P2Y_6_R expression.

Previous studies have demonstrated that P2Y_6_R expression on epithelial cells is upregulated following inflammatory stimuli (9). Therefore, histological lung slides and BAL cells were stained for P2Y_6_R expression. As shown in Figure 1, P2Y_6_R expression was upregulated on lung structural cells in the alveolar space derived from IPF patients whereas no changes were seen on BAL cells (Figure 1; Figure S1 in Supplementary Material).

**Figure 2:**
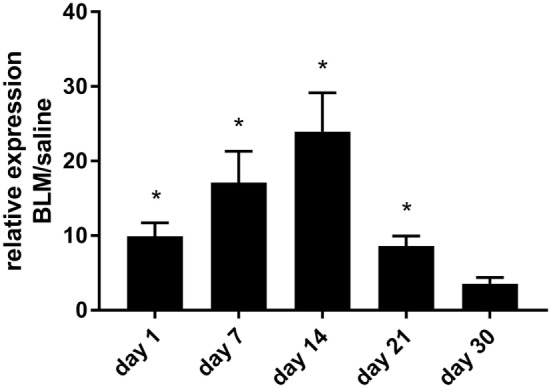
P2Y_6_R expression in bleomycin-induced pulmonary fibrosis. Male C57Bl/6 mice received an i.t. injection of BLM or vehicle on day 0. Animals were killed at the indicated time points and P2Y_6_R expression in lung tissue was analyzed by quantitative RT-PCR (*n* = 3–4 per group) (*p < 0.05).

To confirm the pathophysiological relevance of our findings *in vivo*, we switched to the established mouse model of bleomycin-induced pulmonary fibrosis. As a first approach, P2Y_6_R expression in whole lung tissue was analyzed at different time points after intratracheal injection of bleomycin or vehicle. Thereby, we observed an upregulation of P2Y_6_R expression with a maximum at day 14 after bleomycin exposure with a decline at later time points (Figure 2).

**Figure 3:**
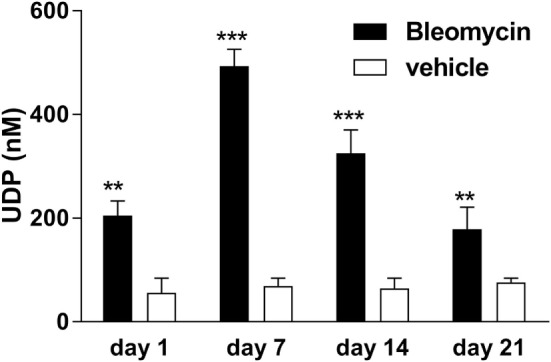
Elevated extracellular uridine-5′-diphosphate (UDP) levels in bleomycin-induced pulmonary fibrosis. Male C57Bl/6 mice received an i.t. injection of BLM or vehicle on day 0. Animals were killed at the indicated time points, and UDP levels were measured in the bronchoalveolar lavage fluid (*n* = 4–6 per group) (**p < 0.01; ***p < 0.001).

Adenosine-5′-triphosphate is abundantly released into the extracellular space under different conditions (4–6, 12, 14). By contrast, much less is known about the potent P2Y_6_R agonist UDP. The concentration of UDP was significantly increased in the BAL fluid after bleomycin exposure with a maximal concentration at day 7 and a decline at later time points (Figure 3).

**Figure 4:**
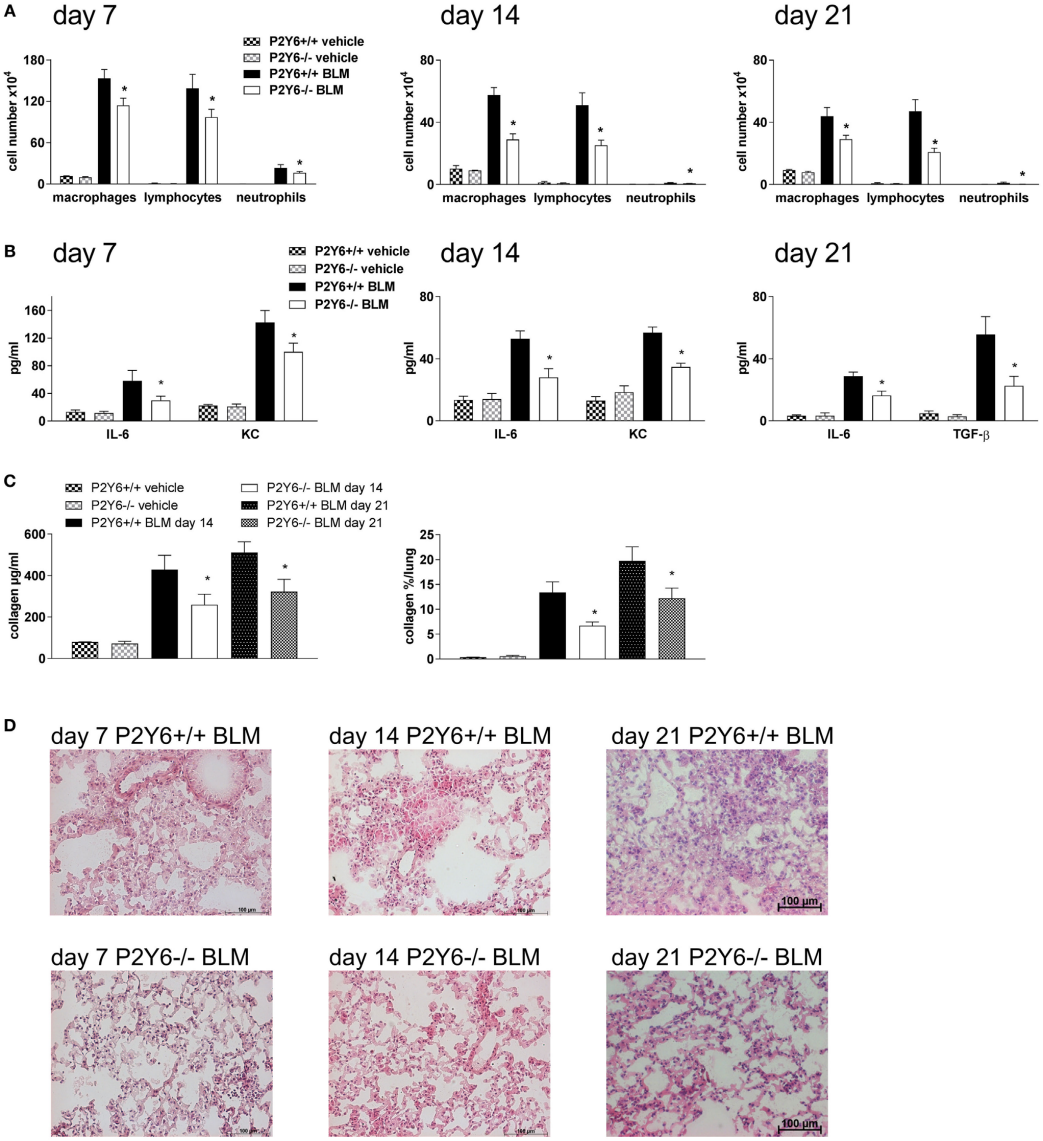
P2Y_6_R in bleomycin-induced pulmonary fibrosis. P2Y_6_R-deficient or wild-type (WT) mice received an i.t. injection of BLM or vehicle on day 0 and were killed 7, 14, or 21 days later. Total and differential cell counts of the BALF were performed **(A)**. BALF cytokines were measured by ELISA **(B)**. Collagen contents of the BALF were quantified by Sircol assay [**(C)**, left panel] or on histological lung slides [**(C)**, right panel]. Representative histological staining of lung slides **(D)**. *n* = 4–14 per group. *p < 0.05 BLM-treated P2Y_6_R-deficient compared to BLM-treated WT animals at the same time point.

To address whether targeting P2Y_6_ receptors might be an approach for the treatment of fibrotic lung disease P2Y_6_R-deficient or WT animals received an intratracheal injection of bleomycin or vehicle at day 0. The degree of inflammation and fibrosis was determined at different time points (7, 14, and 21 days after bleomycin administration). As shown in Figure 4, there was a reduction in pulmonary inflammation at all mentioned time points demonstrated by a reduced number of inflammatory cells in the BAL fluid (Figure 4A) paralleled by a decreased concentration of the pro-inflammatory cytokines IL-6 and keratinocyte-derived chemokine (Figure 4B). In addition, fibrotic tissue remodeling after bleomycin administration was also reduced in P2Y_6_R-deficient demonstrated by reduced collagen content of the BAL fluid and on histological lung slides (Figures 4C,D). Furthermore, BAL fluid levels of the pro-fibrotic cytokine TGF-β was significantly lower in P2Y_6_R-deficient animals (Figure 4B right panel).

**Figure 5:**
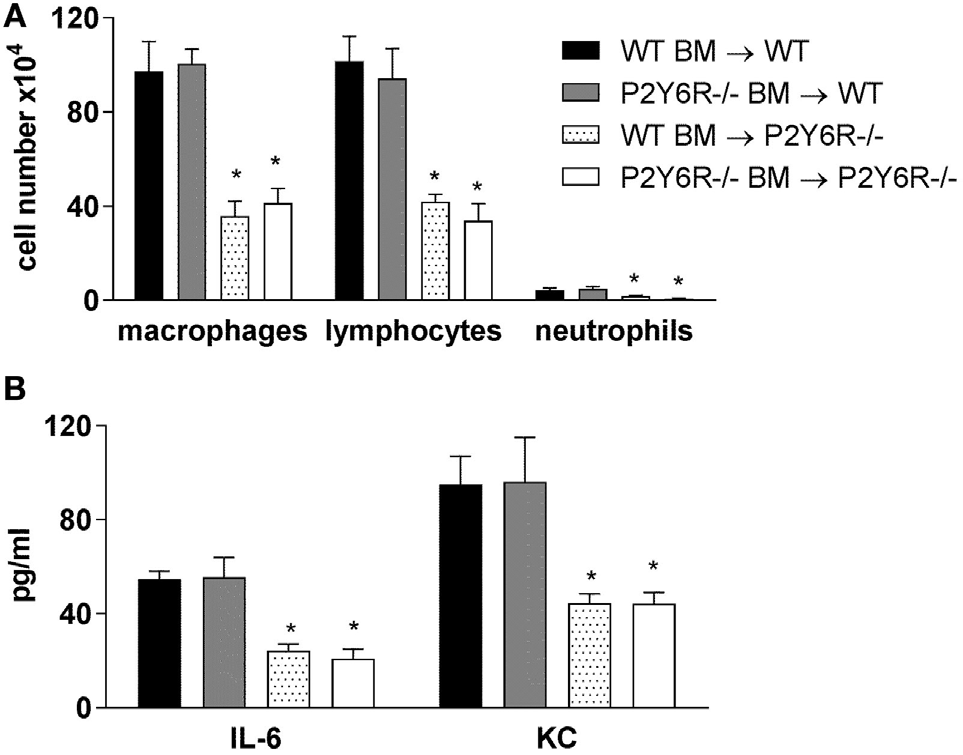
Involvement of P2Y_6_R subtypes on inflammatory and lung structural cells in bleomycin-induced pulmonary fibrosis. The indicated chimeric animals were generated. Mice received an intratracheal application of BLM on day 0. Bronchoalveolar lavage total and differential cell counts were performed, **(A)** and cytokine concentrations were measured by ELISA **(B)** at day 14. *n* = 5–8 per group. *p < 0.05 compared to WT BM → WT or to P2Y_6_R^−/−^ BM → WT.

Next, we sought to determine the relative involvement of P2Y_6_ receptors on hematopoietic and lung structural cells. Therefore, the following bone marrow chimeric mice were generated and exposed to bleomycin: WT BM → WT, P2Y_6_R^−/−^ BM → WT, WT BM → P2Y_6_R^−/−^, and P2Y_6_R^−/−^ BM → P2Y_6_R^−/−^. As shown in Figure 5, the loss of P2Y_6_R expression on lung structural (WT BM → P2Y_6_R^−/−^ and P2Y_6_R^−/−^ BM → P2Y_6_R^−/−^) but not on hematopoietic cells (P2Y_6_R^−/−^ BM → WT) was associated with a significant decrease in bleomycin-induced lung inflammation.

**Figure 6:**
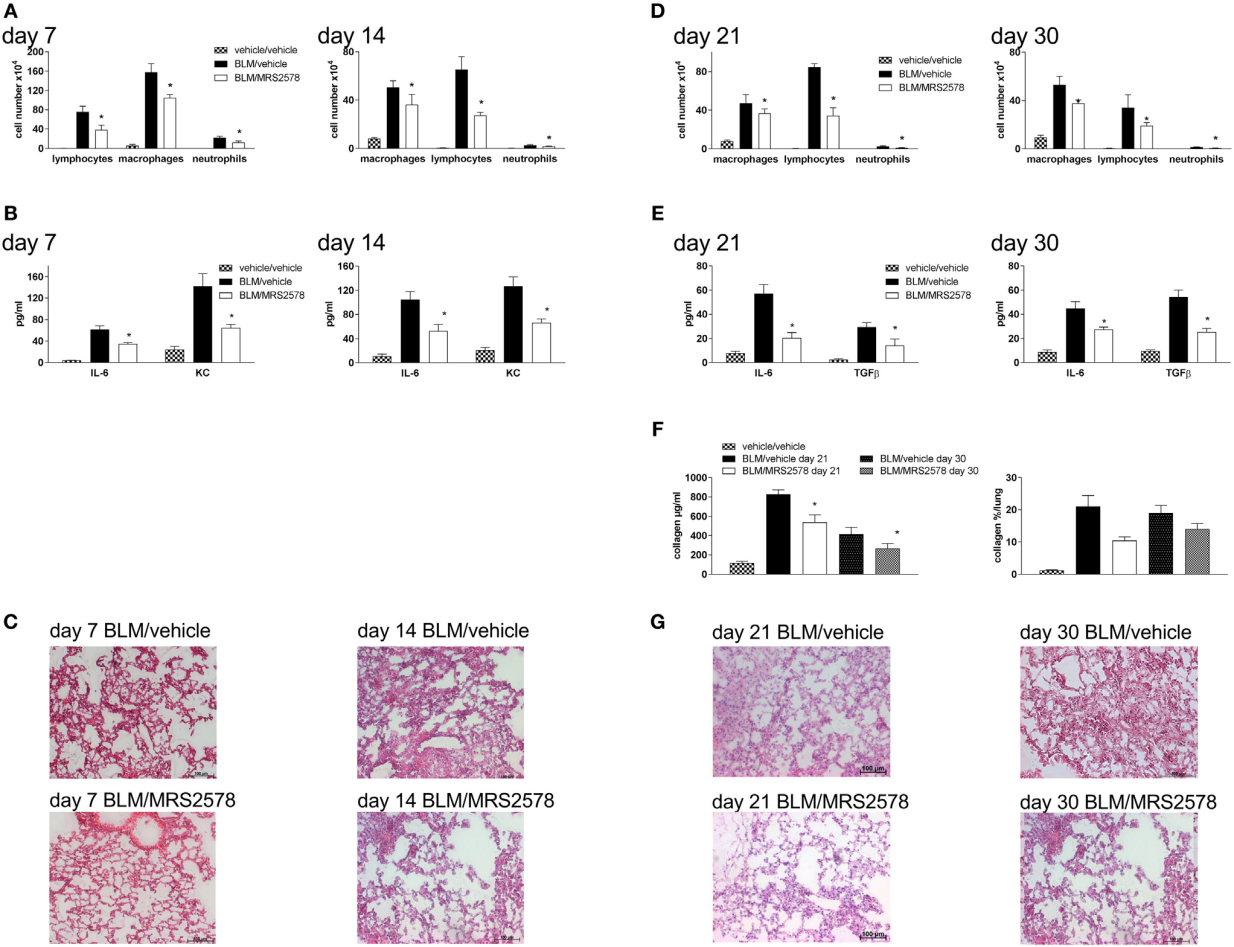
Blocking P2Y_6_R subtypes decreases bleomycin-induced pulmonary fibrosis. Male C57Bl/6 mice received an i.t. application of BLM on day 0 and were treated with the P2Y_6_R antagonist MRS2578 or vehicle according to the prophylactic **(A–C)** or therapeutic **(D–G)** protocol. Total and differential cell counts of the BALF were performed **(A,D)**. BALF cytokines were measured by ELISA **(B,E)**. Collagen contents of the BALF were quantified by Sircol assay [**(F)**, left panel] or on histological lung slides [**(F)**, right panel]. Representative histological staining of lung slides **(C,G)**. *p < 0.05 compared to BLM/vehicle-treated animals at the same time point.

We next questioned whether treatment with a specific P2Y_6_R antagonist would have similar effects as P2Y_6_R deficiency. Therefore, animals were treated with the P2Y_6_R antagonist MRS2578 according to two different protocols: in the prophylactic protocol animals received an intratracheal bleomycin injection on day 0 and were treated with MRS2578 or vehicle after 5 h, 5 days, and 10 days. As shown in Figure 6, treatment with MRS2578 decreased pulmonary inflammation at day 7 and day 14 after bleomycin administration demonstrated by a reduced number of inflammatory cells (Figure 6A) and reduced levels of pro-inflammatory mediators in the BAL fluid (Figure 6B), and on histological lung slides (Figure 6C). To address fibrotic changes during the late phase after bleomycin administration, another protocol (therapeutic protocol) was used in which treatment with MRS2578 or vehicle was started at day 14 for three times a week. Likewise, inflammation (Figures 6D,E,G) and fibrosis (Figure 6F) were decreased in MRS2578-treated animals on day 21 and day 30 after bleomycin exposure.

**Figure 7:**
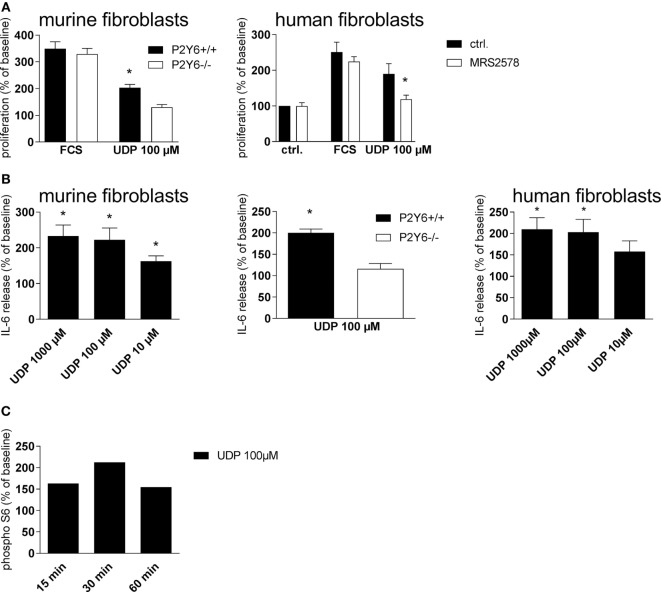
P2Y_6_Rs induce proliferation and cytokine secretion of lung fibroblasts. Primary murine lung fibroblasts were generated from wild-type or P2Y_6_R-deficient animals. The proliferation rate after stimulation with uridine-5′-diphosphate (UDP) was determined [**(A)**, left panel]. Proliferation rate of human primary lung fibroblasts after pre-incubation with the P2Y_6_R antagonists MRS2578 and stimulation with UDP [**(A)**, right panel]. IL-6 release of primary murine [**(B)**, left panel and middle] and human [**(B)**, right panel] lung fibroblasts stimulated with UDP was measured by ELISA. S6 phosphorylation after stimulation with UDP was measured by Western Blot at the indicated time points. One representative experiment out of three is shown **(C)**. *n* = 3–5 per group (*p < 0.05).

In pursuit of the mechanisms involved in P2Y_6_R-induced progression of pulmonary inflammation and fibrosis, *in vitro* experiments with human and murine cells were performed. To check the involvement of P2_6_R subtypes in regulating the proliferation rate of lung fibroblasts, primary murine lung fibroblasts were treated with UDP or vehicle as a negative control and cell proliferation was assessed after 48 h. Thereby, UDP increased fibroblast proliferation (Figure 7A, left panel), an effect which could not be observed with cells derived from P2Y_6_R-deficient animals. Similar results were obtained with primary human lung fibroblasts (Figure 7A, right panel). Next, we questioned whether P2_6_R signaling is also involved in IL-6 production by lung fibroblasts, a cytokine involved both in acute inflammation and tissue remodeling (24). UDP dose dependently increased IL-6 production by murine (Figure 7B, left panel) and human (Figure 6B, right panel) fibroblasts *via* activation of P2Y_6_R subtypes (Figure 7B, middle). Activation of the S6–mTOR pathway in fibroblasts plays an important role in the pathogenesis of lung fibrosis (25). Therefore, we next determined the effect of UDP on Akt and S6 phosphorylation showing that stimulation with UDP resulted in a transient activation of pS6 (Figure 7C).

## Discussion

Signaling *via* purinergic receptors has been demonstrated to be crucial for the pathophysiology of various lung disorders including pulmonary fibrosis (8, 14). As we were previously able to show that the activation of P2Y_6_ receptors is associated with inflammation and tissue remodeling in an animal model of allergic lung inflammation, we were interested whether this purinergic receptor subtype is also involved in the pathogenesis of fibrotic lung diseases (9).

We observed an upregulation of pulmonary P2Y_6_R expression in animals with bleomycin-induced lung fibrosis. Immunohistochemical staining of human lung tissue samples showed an upregulation of P2Y_6_R expression on lung structural cells in patients suffering from IPF though samples from only few patients were available which has to be considered as a limitation of our study. Nevertheless, these results are in line with previous studies showing increased P2Y_6_R expression on resident cells under inflammatory conditions (9, 17, 26). Interestingly, as bleomycin-induced pulmonary fibrosis is known to be self-limiting there was a decline in P2Y_6_R expression at later time points (27). In addition to P2Y_6_R expression, the concentration of UDP which is the strongest naturally occurring agonist at P2Y_6_ receptors was also elevated following bleomycin administration. While numerous studies demonstrated increased extracellular ATP levels in inflamed tissues, much less is known about the release of UPD into the extracellular space in the context of inflammation or tissue remodeling (4, 5, 12, 28).

The degree of inflammation and fibrosis following bleomycin administration was attenuated in P2Y_6_R-deficient animals compared to WT animals. Similar results were obtained when WT animals were treated with the P2Y_6_R antagonist MRS2578. While the role of P2Y_6_ receptors in the context of acute inflammation, e.g., acute allergic airway, intestinal or vascular inflammation, has been studied extensively much less is known about the role of this receptor subtype in tissue remodeling and fibrosis, though in an animal model of chronic allergic airway inflammation, P2Y_6_R activation was also associated with airway remodeling (9, 17, 26). In accordance, another study demonstrated UDP and ATP release by cardiomyocytes triggered by mechanical stretch leading to P2Y_6_R-dependent cardiac fibrosis (19).

An upregulation of P2Y_6_R expression in lung structural derived from IPF patients and animals after bleomycin administration was observed. In addition, experiments with chimeric animals revealed that P2Y_6_R expression on lung structural rather than on immune cells was responsible for P2Y_6_R-induced inflammation and fibrosis in accordance with previous studies (9, 17). Hence, *in vitro* experiments with lung fibroblasts were performed in pursuit of the involved mechanisms. Thereby, we were able to show that UDP increased the proliferation rate of human and murine lung fibroblasts *via* activation of P2Y_6_ receptors. Similar results have been made previously with different cell types though the effect of P2Y_6_R activation on the proliferation rate of fibroblasts has not been studied in detail, yet (29, 30).

Apart from the synthesis and the release of extracellular matrix components, lung fibroblasts have also been demonstrated to exert immune-regulatory properties. Stimulation of primary lung fibroblasts resulted in increased production of the pro-inflammatory cytokine IL-6 which is associated with tissue remodeling (24, 31–33). This is in a line with previous work which demonstrated the importance of P2Y_6_-triggered release of pro-inflammatory cytokines by epithelial cells for the pathogenesis of bronchial asthma or inflammatory bowel disease (9, 17).

Stimulation with UDP increased S6 phosphorylation in lung fibroblasts indicating the activation of the S6–mTOR pathway which has been shown to contribute to the pathogenesis of pulmonary fibrosis (25, 34, 35). Apart from this, mTOR activation has also been observed after stimulation with ATP suggesting the importance of different purinergic receptor subtypes (36).

In summary, we could show the involvement of the purinergic receptor subtype P2Y_6_ into the pathogenesis of pulmonary fibrosis in human and mice *via* several mechanisms. However, further studies are needed to investigate if targeting P2Y_6_ receptors might be a new approach for the treatment of pulmonary fibrosis in humans.

## Ethics Statement

Animal experiments were carried out in accordance with national regulations. All experiments were approved by the local animal ethics committee (Regierungspräsidium Freiburg). The use of Patient materials was approved by the ethics committee at the University Medical Center Freiburg. All subjects gave written informed consent in accordance with the Declaration of Helsinki.

## Author Contributions

Experiments were planned, performed, and analyzed by TM, SF, RV, SC, TG, CKA, and ERL. HK-Q, GZ, and MI helped with the analysis of the experiments. The manuscript was written by TM, HK-Q, GL, DF, FV, BR, J-MB, MB, and MI. The manuscript was read and approved by all the authors.

## Conflict of Interest Statement

The authors declare that the research was conducted in the absence of any commercial or financial relationships that could be construed as a potential conflict of interest.
