# Supplementary material for: P2Y6 Receptor Activation Promotes Inflammation and Tissue Remodeling in Pulmonary Fibrosis
Source: Front Immunol. 2017 Aug 22;8:1028. doi: 10.3389/fimmu.2017.01028 (PMC5572280; doi:10.3389/fimmu.2017.01028)
Supplement: Supplementary file 1 [file image_1.pdf]

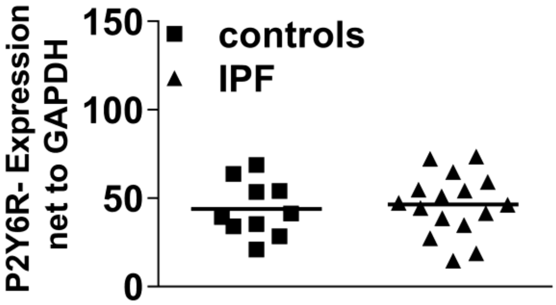

Supplementary figure. P2Y$_6$R expression in idiopathic pulmonary fibrosis.

Expression of P2Y$_6$ receptor subtypes on BAL cells derived from IPF patients (n= 16) and healthy individuals (n=10) was determined by quantitative RT-PCR.
